# Hazediff: A training-free diffusion-based image dehazing method with pixel-level feature injection

**DOI:** 10.1371/journal.pone.0329759

**Published:** 2025-10-28

**Authors:** Xiaoxia Lin, Zhengao Li, Dawei Huang, Wancheng Feng, XinJun An, Lin Sun, Niuzhen Yu, Yan Li, Chunwei Leng

**Affiliations:** 1 College of Intelligent Equipment, Shandong University of Science and Technology, Taian, China; 2 Hanqing Data Consulting Co., Ltd., Zibo, China; Bayer Crop Science United States: Bayer CropScience LP, UNITED STATES OF AMERICA

## Abstract

In the current environmental context, significant emissions generated by industrial and transportation activities, coupled with an unreasonable energy structure, have resulted in recurrent haze phenomena. This consequently leads to degraded image contrast and reduced resolution in captured images, significantly hindering subsequent mid- and high-level visual tasks. These technical challenges have positioned image dehazing as a pivotal research frontier in computer vision. Nevertheless, current image dehazing approaches exhibit notable limitations. Deep learning-based methodologies demand extensive paired hazy-clean training datasets, the acquisition of which remains particularly challenging. Furthermore, synthetically generated data frequently exhibit marked disparities from authentic scenarios, thereby limiting model generalizability. Despite diffusion-based approaches demonstrating superior image reconstruction performance, their data-driven implementations face comparable limitations. To overcome these challenges, we propose HazeDiff: a training-free dehazing method based on the Diffusion model. This method provides a novel perspective for image dehazing research. Unlike existing approaches, it eliminates the need for hard-to-get paired training data, reducing computational costs while enhancing generalization. This not only reduces computational costs but also improves the generalization ability and stability on different datasets. Ultimately, it ensures that the dehazing restoration results are more reliable and effective. The Pixel-Level Feature Inject (PFI) we proposed is implemented through the self-attention layer. It integrates the pixel-level feature representation of the reference image into the initial noise of the dehazing image, effectively guiding the diffusion process to achieve the dehazing effect. As a supplement, the Structure Retention Model (SRM) incorporated in Cross-attention performs dynamic feature enhancement through adaptive attention re-weighting. This ensures the retention of key structural features during the restoration process while reducing detail loss. We have conducted comprehensive experiments on both real-world and synthetic datasets.Experimental results demonstrate that HazeDiff surpasses state-of-the-art dehazing methods, achieving higher scores on both no-reference (e.g., NIQE) and full-reference (e.g., PSNR) evaluation metrics. It shows stronger generalization ability and practicality. It can restore high-quality images with natural visual features and clear structural content from low-quality hazy images.

## Introduction

**Fig 1:**
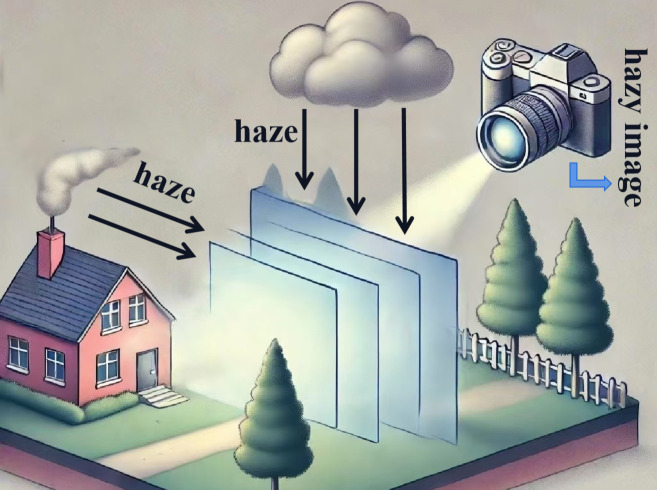
Haze in the real world makes image quality worse. This shows we need effective haze removal.

In the various applications of computer vision, image dehazing is an important and challenging research area. Whether in daily image capturing, security monitoring, or key fields like autonomous driving assistance, clear images are the foundation for accurate analysis and decision-making. However, airborne particles such as dust and smoke are prevalent in the atmosphere, exhibiting strong light absorption and scattering properties. This phenomenon results in haze-obscured images with severely degraded contrast and clarity, substantially impairing their utility for tasks such as object detection and scene recognition. As shown in Fig 1.

Currently, the main dehazing methods are generally based on deep learning and diffusion models. Deep learning-based dehazing methods, such as Dehazenet [1], dehaze images by learning the medium transmission in the haze degradation model; FFA-Net [2] introduces a feature attention mechanism (FAM) into the dehazing network; GDN [3]; and MSBDN [4] propose a multi-scale enhanced dehazing network based on the U-Net architecture, presenting an end-to-end trainable convolutional neural network. Dehamer [5] generates high-quality dehazed images through convolutional blocks and multi-scale residual blocks. These methods typically require considerable effort and resources to train models with large amounts of data, but obtaining suitable training data is quite challenging. This is because it is nearly impossible to obtain paired images of the same scene in both hazy and clear conditions in the real world. Additionally, due to the lack of effective use of prior knowledge, the stability and consistency of dehazing results based on deep learning methods are difficult to ensure.Diffusion model-based dehazing methods can leverage the prior knowledge of large pre-trained models, demonstrating unique advantages in dehazing tasks. However, some studies, when optimizing dehazing effects by training additional plugins, again face challenges such as the scarcity of real hazy scene training data and the mismatch between synthetic and real data distributions. For example, RSHazeDiff [6] applies contrastive constraints during Fourier-perceived condition diffusion model training, while MonoWAD [7] uses hazy-clear paired features during the training phase. Through convolutional layers and a quantization process, it generates weather reference features.

In dehazing methods that require training, the datasets used for model training are mainly constructed in two ways. First, most studies simulate hazy images by artificially adding haze to clear-day images to create synthetic hazy data. Second, some researchers use haze machines in real-world scenes to directly capture hazy images along with their corresponding real clear images. However, these artificially created hazy images have fundamental differences from images captured in real hazy conditions. These differences limit the generalization ability of deep learning models trained on these datasets. As a result, when the model encounters entirely new, untrained real hazy images, it lacks targeted learning of the specific features of these true hazy images. The dehazing model cannot accurately extract and process haze information in the images, making it difficult to effectively remove the haze. Consequently, dehazed images exhibit deficiencies in clarity, color fidelity, and detail preservation, thereby diminishing the reliability and practicality of such models in real-world scenarios.

We have observed significant progress in using reference images [8–11] to guide diffusion models in image synthesis, editing, and stylization. However, image synthesis and editing often struggle to achieve transformations in both content and tone simultaneously, while stylization tends to disrupt the structure of the original hazy image. Despite this, using reference images to guide diffusion models still holds value for dehazing, as it can effectively reference two real datasets (one normal dataset and one hazy dataset).

Against this backdrop, we propose an innovative HazeDiff framework. This framework uses reference images to guide the diffusion model for dehazing, without the need for complex training processes. The core idea is to cleverly integrate the distribution features of clear-weather images into hazy images while preserving the original structure of the hazy image, thereby achieving high-quality dehazing. Specifically, we select a clear-weather image as a reference and use a specific algorithm to fuse the latent noise of the two images, obtaining the initial noise for the dehazed image and performing a reverse diffusion process. In this process, we introduce a PFI method. This method feeds the hazy data into a self-attention mechanism, and by replacing the corresponding parts with key-value pairs from the reference image, it successfully transfers the pixel-level features of the reference image to the hazy image. Furthermore, to ensure that the model not only preserves the original structure of the hazy image but also accurately focuses on key regions and details during the dehazing process, we design an attention-based SRM, which effectively overcomes the problem of blurred structural details in the generated image.

We conduct comprehensive experiments on both reference-free real-world datasets and synthetic datasets with corresponding clear references. The experimental results show that, compared to existing advanced dehazing methods, HazeDiff achieves the best performance in dehazing synthetic hazy images, artificially generated hazy images, and real-world hazy images. It demonstrates stronger generalization ability and practicality, successfully recovering high-quality images with natural visual features and clear content structure from low-quality, blurry hazy images.

In summary, our contributions can be listed as follows:

- We propose HazeDiff, a novel diffusion-based dehazing framework. Using a training-free strategy, it achieves accurate dehazing while effectively avoiding the challenges of hazy data.
- We propose a pixel injection method, PFI, which better guides the content image to achieve pixel-level transformation by replacing the key and value in the model’s self-attention.
- We propose an attention-based object edge retention module, SRM, which helps the model focus on the key areas and details of the hazy image, solving the problem of losing original structural details during the generation process.
- We conducted extensive experiments on real hazy datasets. The results show that the method proposed in this paper significantly outperforms previous methods.

## Related work

### Deep learning-based image dehazing methods

Deep learning has been widely applied in fields such as computer vision, image classification, and object detection, and is gradually being applied to image dehazing tasks. The Dehazenet [1] dehazing network includes a feature extraction layer combined with traditional handcrafted features, a multi-scale mapping layer, a local extrema layer, and a nonlinear regression layer, which learns the medium transmittance in the haze degradation model to perform dehazing. IA-YOLO [12] relies on a large amount of high-quality labeled data for training. Through multi-scale feature fusion, it utilizes deep-layer features (containing high-level semantic information) to help restore the details blurred by haze. FFA-Net [2] introduces the Feature Attention Mechanism (FAM) into the dehazing network to handle different types of information. GDN [3] proposes an end-to-end trainable convolutional neural network that introduces attention-based multi-scale estimation to guide single-image dehazing. Dehamer [5] uses Transformer to establish long-range dependencies, guided by haze density, to generate high-quality dehazed images through convolutional blocks and multi-scale residual blocks. MSBDN [4] proposes a multi-scale enhanced dehazing network based on the U-Net architecture, with a dense feature fusion method that uses boosting strategies and back-projection techniques to enhance feature fusion. The decoder gradually restores the dehazed image, improving dehazing performance under specific axioms.

Deep learning-based methods often require training a completely new architecture from scratch. On one hand, these methods lack prior knowledge, making it difficult to guarantee effective dehazing. On the other hand, they commonly face the issue that model performance depends on large amounts of hazy scene data for training. Factors such as data quality, quantity, and diversity all impact the model’s generalization ability, and paired hazy and clear weather data from the same scene are nearly impossible to obtain. Some methods add haze to clear weather data and use these augmented data for training. However, the distribution of haze-added data differs fundamentally from that of real hazy scene data, which may lead to a decrease in the model’s adaptability to different hazy conditions in practical applications.

### Diffusion model-based image dehazing methods

Diffusion-based generative models [13,14] gradually introduce noise to obtain the latent noise of an image, and then iteratively denoise from the noise distribution to recover a clear image. This approach effectively leverages the prior knowledge of large models. Some works choose to train a plug-in, but when training the plug-in, they encounter the same issue of difficulty in obtaining training data, as well as the disparity between haze-added data and real data. During the process of Frequency [15] training the dehazing framework based on the conditional diffusion model, these paired data are used to learn the parameters of the model, enabling the model to learn the mapping relationship from hazy images to clear images and thus achieve the dehazing function. The RSHazeDiff [6] model training also requires using coarse recovery results and clear real image patches for contrastive constraints during the Fourier perceptual condition diffusion model training. MonoWAD [7] in the training phase, it receives paired clear-hazy features. Through the convolution layer and the quantization process, it generates weather-reference features. It is trained with the clear knowledge recalling (CKR) loss composed of the clear knowledge embedding (CKE) loss and the weather-invariant guiding (WIG) loss, enabling it to remember the knowledge of clear weather and generate reference features for different weather conditions.

Some studies on image restoration using conditional diffusion models, in addition to requiring a large amount of training data, may also have limitations in the dehazing application scenarios due to their reliance on physical models. Diff-Retinex [16] puts forward a low-light image enhancement method based on physical interpretability and generative diffusion models. It transforms the low-light image enhancement problem into Retinex decomposition and conditional image generation problems. In [17], a unified conditional framework based on diffusion models for image restoration is proposed. Additionally, there is a risk of significantly damaging the original structure of the image. Dehaze-DDPM [18] proposed an innovative two-stage image dehazing model, dehazedDPM. In the first stage, the model introduces a physical model to perform initial processing of the image, estimating key parameters to generate a new image. In the second stage, the model uses the output of the first stage as a condition to guide the diffusion model for sampling. A visual-textual dual-encoder was constructed to map abstract text entities to specific image regions. This type of model has limited understanding of text semantics, which may lead to poor consistency between the generated image and the text semantics and also poses a significant risk of damaging the original structure of the image.

We avoid these limitations from a new perspective.We use a reference image to guide the diffusion model without training. This improves the model’s generalization ability and makes it better suited to the complexity of real hazy scenes.

## Research methods

Based on the Diffusion Model, our training-free strategy focuses on the pixel and structural features in the reference image and hazy image to achieve effective dehazing. In section 3.1, we introduce the background of Stable Diffusion; In section 3.2, we describe the overall pipeline of HazeDiff; In section 3.3, we explain the Pixel-Level Feature Inject method and its implementation; In section 3.4, we provide an overview of the design and implementation of the Structure Retention Model to preserve the original structure details in hazy images.

### Preliminary: Denoising diffusion implicit models

The Denoising Diffusion Implicit Model is a non-Markovian latent variable model that achieves efficient generation through deterministic sampling trajectories. Its core framework consists of two key processes:

Forward Diffusion Process: The input image *x*_0_ is encoded into a latent variable $z_{0}=E(x_{0})$, and noise is gradually added via a predefined scheduling mechanism. The mathematical formulation is:

$$q(z_{t}|z_{0})=𝒩\left(z_{t};\sqrt{\alpha_{t}}z_{0},(1-\alpha_{t})I\right)$$ (1)

Here, $\alpha_{t}$ (t=0,1,…,T) is a monotonically decreasing noise scheduling parameter that controls the rate of noise accumulation, and *T* denotes the total diffusion steps. By directly linking the initial latent variable *z*_0_ to any intermediate state *z*_*t*_, this process supports non-Markovian jumps, enabling flexible trajectory design.

Reverse Denoising Process: The reverse process follows a deterministic generation path, reconstructing the latent variables step-by-step using a noise-prediction model $\epsilon_{\theta }$. Specifically, the update from *z*_*t*_ to *z*_*t*−1_ is governed by:

$$z_{t-1}=\sqrt{\alpha_{t-1}}\underset{\text{estimated initial latent variable }z_{0}}{\underbrace{\left(\frac{z_{t}-\sqrt{1-\alpha_{t}}\epsilon_{\theta }(z_{t},t)}{\sqrt{\alpha_{t}}}\right)}}+\sqrt{1-\alpha_{t-1}-\sigma_{t}^{2}}\cdot \epsilon_{\theta }(z_{t},t)$$ (2)

The parameter $\sigma_{t}^{2}$ adjusts stochasticity (typically set to 0 for fully deterministic generation). Unlike traditional approaches, this reverse process eliminates the need to learn covariance matrices, relying solely on the noise-prediction model $\epsilon_{\theta }$ to drive the dynamics.

### HazeDiff pipeline

**Fig 2:**
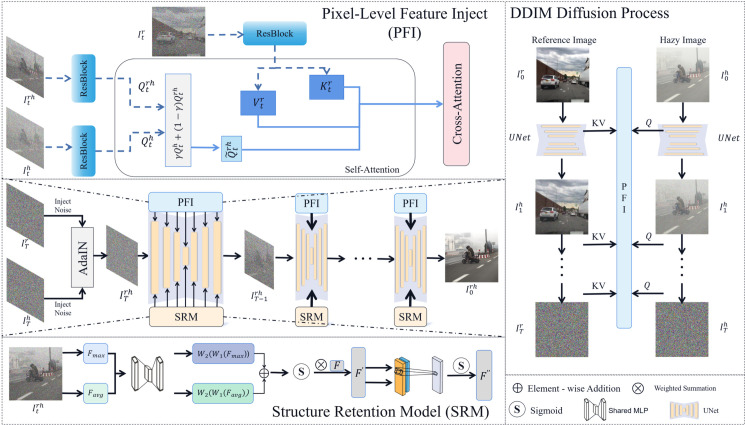
The overall framework of the method in this paper.

The overall architecture of the proposed method is shown in Fig 2. Inspired by the work of [11,19,20], we using the U-Net [21] architecture and leveraging the pre-trained model of Stable Diffusion. At time step 0, we invert the reference image ($I_{0}^{r}$) and the hazy image ($I_{0}^{h}$) into their respective latent noises. Then, we combine the two latent noises using AdaIN [22] to initialize the initial noise $I_{T}^{rh}$ of the dehazed image, and perform the reverse diffusion process on this initial noise.

DDIM Diffusion Process (right diagram): The hazy image and reference image are separately mapped to the latent noise space through the DDIM Diffusion Process. This produces their corresponding noise images $I_{0}^{h}$ and $I_{0}^{r}$.

The overall framework diagram (middle part of the left diagram) shows the proposed image dehazing workflow:

1. Initial noise generation for dehazed image: Adaptive instance normalization (AdaIN) combines noise $I_{T}^{h}$ from the hazy image with noise $I_{T}^{r}$ from the reference image. This produces initial noise $I_{T}^{rh}$ for the dehazed image, preserving the hazy image’s content structure while incorporating the reference image’s color distribution.

2. Reverse diffusion process: Starting from the initial noise of the dehazed image, we execute a reverse diffusion process. During this process, pixel-level features from the reference image are injected through the following operations: Pixel-Level Feature Inject (upper part of the left diagram): In the self-attention layer, the Key $K_{t}^{h}$ and Value $V_{t}^{h}$ of the hazy image are replaced with the corresponding parts ($K_{t}^{r}$, $V_{t}^{r}$) from the reference image. The Query $Q_{t}^{h}$ of the hazy image is mixed with the Query $Q_{t}^{r}h$ of the dehazed image to retain content structure. Structure Retention Model (lower part of the left diagram): The module combines channel attention and spatial attention mechanisms. It dynamically weights feature channels through global pooling operations and a shared MLP. Meanwhile, convolution integrates channel-pooled features to enhance key spatial regions.

### Pixel-level feature inject

Inspired by some generative methods [11,23,24], we found that injecting pixel-level features into the *k* and *v* of self-attention can better guide the content image to achieve pixel-level transformations. The specific operation of self-attention is as follows:

$$Q=W_{Q}(\phi ),\;K=W_{K}(\phi ),\;V=W_{V}(\phi )$$ (3)

$$\phi_{out}=Attn(Q,K,V)=softmax\left(\frac{QK^{⊤}}{\sqrt{d}}\right)\cdot V$$ (4)

Here, *d* denotes the dimension of the projected query, W(·) is the projection layer, and *ϕ* is the feature after the residual block.

We adjust the self-attention mechanism by using the features extracted from the $I_{0}^{r}$ as conditions. Specifically, in the self-attention, we replace the keys and values in $I_{t}^{rh}$ with the keys and values from $I_{t}^{r}$ at the same time step *t*, without requiring any textual supervision. As a result, the dehazed image $I_{0}^{rh}$ retains the original structure of $I_{0}^{h}$ while effectively removing the haze and injecting the pixel distribution of normal weather.

As shown in Fig 2, in order to achieve this goal, at each noisy time step t=(0,1,…,T), we collect the query features $Q_{t}^{h}$ from the $I_{0}^{h}$ image and the key features $K_{t}^{r}$ and value features $V_{t}^{r}$ from the $I_{0}^{r}$ image. During the reverse denoising process, we inject the collected key features $K_{t}^{r}$ and value features $V_{t}^{r}$ from the $I_{0}^{r}$ image into the self-attention layer, replacing the original key features $K_{t}^{rh}$ and value features $V_{t}^{rh}$. This transfers the clear, haze-free pixel distribution from the $I_{0}^{r}$ to the latent representation $I_{t}^{rh}$ of $I_{0}^{rh}$.

In Stable Diffusion, the query vector *Q* determines the range or object that needs to be focused on and queried. We aim to perform dehazing on hazy images with varying levels of haze by controlling *Q*. Therefore, during the denoising process, we mix the query $Q_{t}^{rh}$ from the latent representation of $I_{t}^{rh}$ with the query features $Q_{t}^{h}$ from $I_{t}^{h}$. The operation of the self-attention block can be expressed as follows:

$${\tilde{Q}}_{t}^{rh}=\gamma \times Q_{t}^{h}+(1-\gamma )\times Q_{t}^{rh}$$ (5)

$$\phi_{out}^{rh}=Attn\left({\tilde{Q}}_{t}^{rh},K_{t}^{r},V_{t}^{r}\right)=softmax\left(\frac{{\tilde{Q}}_{t}^{rh}{\left(K_{t}^{r}\right)}^{⊤}}{\sqrt{d}}\right)\cdot V_{t}^{r}$$ (6)

Here, *γ* is the mixing degree parameter, with a value range of [0,1]. We adjust the dehazing strength by changing the value of *γ*. Specifically, the higher the value of *γ*, the weaker the dehazing effect, and more content from the hazy image, including structure and pixel-level haze, will be preserved. Conversely, the lower the value of *γ*, the stronger the dehazing effect, but it may also cause the dehazed image to lose more key details of the original content. Ablation studies (section 4.4) demonstrate that *γ* = 0.5 balances haze removal and structural preservation.

### Structure retention model

During the dehazing process, preserving the original structural details in the input is challenging. Therefore, during the dehazing process, important features such as object contours and key textures in the hazy image should be assigned relatively higher weights, while the weights of areas that are heavily affected by haze and less helpful for content understanding should be appropriately reduced.

Some Diffusion-based image editing tasks [23,25] propose that the cross-attention map directly influences the generation results. Inspired by [26–28], we designed a SRM within the Cross Attention framework. Leveraging the SRM’s ability to adaptively focus and filter features across both channel and spatial dimensions, we further optimize the feature representations involved in the Cross Attention process, ensuring better preservation of key structural elements such as object contours and relative spatial relationships in hazy images during the dehazing process. For example, when processing hazy images containing various objects such as buildings and vehicles, structural information like the shape of buildings and the form of vehicles, which are typically prone to blurring or loss, is more effectively preserved in the final dehazed image through SRM’s attention and enhancement across both channel and spatial dimensions.

The SRM combines channel attention and spatial attention to adaptively focus on haze-relevant features and structural regions. Channel attention prioritizes haze-relevant features by weighting channels based on their importance, while spatial attention focuses on structural regions (e.g., edges and contours) to preserve key details during dehazing.

As shown in Fig 2, we first apply global average pooling and global max pooling operations to the input content structure feature map (i.e., hazy image) $F\in R^{C\times H\times W}$, where *C* represents the number of channels, *H* is the height of the feature map, and *W* is the width of the feature map:

$$F_{avg}=\frac{1}{H\times W}\sum_{i=1}^{H}\sum_{j=1}^{W}F_{c}(i,j)$$ (7)

$$F_{max}=\max_{i=1}^{H}\max_{j=1}^{W}F_{c}(i,j)$$ (8)

We obtain a *C*-dimensional vector $F_{avg}$ and another *C*-dimensional vector *F*_*max*_, where *F*_*c*_(*i*,*j*) represents the pixel value at position (*i*,*j*) in channel *c*.

Then, these two vectors are passed through a shared multilayer perceptron (MLP), where the shared MLP has two layers, with the first layer weights *W*_1_ and the second layer weights *W*_2_, to obtain the channel attention weights. The process of applying the channel attention-weighted feature map is shown as follows:

$$F^{\prime }=(\sigma (W_{2}(W_{1}F_{avg})+W_{2}(W_{1}F_{max}))⊗F$$ (9)

After the feature map $F^{\prime }$is weighted by the channel attention weights, it undergoes average pooling and max pooling along the channel dimension in the spatial attention mechanism to obtain feature maps. These maps are then concatenated, convolved, and processed to obtain a 1-channel feature map. After passing through a sigmoid function, the feature map represents the attention target location information, highlighting the key regions of the hazy image features in the spatial dimension. The process is shown as follows:

$$F^{\prime \prime }=\sigma (f^{7\times 7}([\frac{1}{C}\sum_{c=1}^{C}F_{c}^{\prime },\max_{c=1}^{C}F_{c}^{\prime }]))⊗F^{\prime }$$ (10)

## Experiment

This paper will first present detailed experimental data and evaluation metrics. Then, we will compare our method with several recent dehazing methods to verify its effectiveness. After that, we will conduct an ablation study on the proposed method to further demonstrate its necessity.

### Datasets

We conducted comprehensive experiments using the publicly available large-scale dataset RESIDE [29] (REalistic Single Image DEhazing) to evaluate the effectiveness of our proposed dehazing model. This dataset is divided into subsets with different purposes (training or evaluation) or sources (indoor or outdoor) based on various data sources and image content. In the experiments, we selected synthetic hazy images and their corresponding clear images from the ITS (Indoor Training Set) subset, as well as highly challenging real-world hazy images (without corresponding clear images) from the RTTS (Real-world Task-Driven Testing Set) subset, for dehazing experiments. Additionally, we evaluated the performance and generalization ability of our method using non-homogeneous hazy images created with haze machines from the NH-HAZE dataset [30]. The specific details of the dataset are shown in Table 1.

**Table 1:** The specific details of the dataset used in the experiment.

Dataset	Subset	Data volume	Haze type	Scene	Clear pairing picture
RESIDE	ITS	13990 pairs	Synthetic uniform haze	Indoor	✓
RESIDE	RTTS	4322(Only haze map)	Real haze	Outdoor	×
NH-HAZE	-	55 pairs	True non-uniform haze	Indoor + Outdoor	✓

### Evaluation metrics

For the dehazing results of synthetic data selected from the ITS subset, we used widely adopted full-reference evaluation metrics in dehazing tasks: Peak Signal-to-Noise Ratio (PSNR) and Structural Similarity Index (SSIM) [31]. However, synthetic hazy images and real hazy images have fundamental differences in haze distribution, and real hazy data lack clean, clear images as references. Therefore, in addition to PSNR and SSIM, we adopted no-reference metrics, including the Naturalness Image Quality Evaluator (NIQE) [32] and the Blind/Referenceless Image Spatial Quality Evaluator (BRISQUE) [33], to evaluate the dehazing results of real hazy images selected from the RTTS subset. These no-reference metrics complement the limitations of PSNR and SSIM. Additionally, we used object detection performance on dehazed images as a task-specific no-reference evaluation standard, employing a task-driven evaluation approach to assess the effectiveness of the dehazing algorithm.

### Implementation details

The experiment was conducted on an Ubuntu 18.04 computing platform with an NVIDIA RTX4090 GPU that has 24GB of memory. In terms of software environment, to improve experimental efficiency and code maintainability, we used CUDA version 11.1, Python version 3.8.5, and PyTorch version 1.11.0. During the experiment, most of the configurations were set to default, with adjustments made only to some key parameters.

DDIM [13] inversion steps were set to 50, and the number of steps for saving features was also set to 50. The downsampling factor was set to 8. The hyperparameter *γ*, which controls query retention, was selected between 0 and 1 based on an ablation study to find the optimal value. The layers where attention features were injected are layers 6, 7, 8, 9, 10, and 11. All experiments were repeated 5 times with different random seeds to ensure stability. The reported results are averaged values, with standard deviations below 0.5% for all metrics.

### Experimental results

In this section, we compare the proposed method with existing advanced dehazing methods and conduct experimental analysis. These methods include IA-YOLO [12], FFA-Net [2], GDN [3], Dehamer [5], and MSBDN [4]. Among them, except that IA-YOLO uses the atmospheric scattering model, the remaining models are trained using the synthetic hazy images in the RESIDE [29] dataset and their corresponding real-clear images.In 4.3.1, we will perform a qualitative comparison of the dehazed images obtained using the aforementioned methods and our method. In 4.3.2, to validate the results of the quantitative comparison analysis, we will use the evaluation metrics widely used in dehazing performance assessment, as mentioned earlier, for a quantitative comparison.In 4.3.3, to further verify the effectiveness and applicability of the proposed method in the real world, we compare the mean Average Precision (mAP) obtained from YOLO [34] detection on the dehazed RTTS hazy dataset with other related works in hazy weather object detection.

#### A qualitative comparison of defogged foggy images under various circumstances.

In this part, we will present the dehazing results produced by the aforementioned recent methods and the proposed method, and conduct a qualitative comparative analysis. We show the results obtained from artificially simulated hazy images and real-world hazy images.

**Fig 3:**
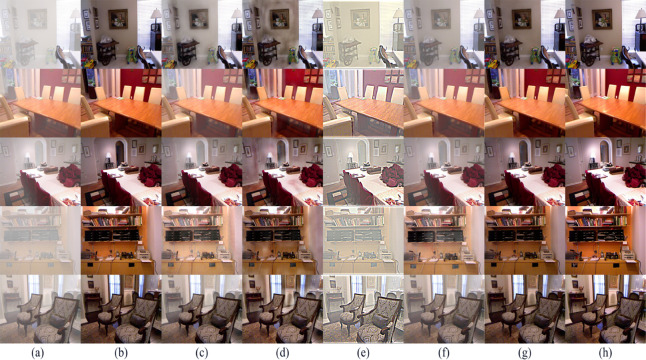
Subjective comparison on the ITS synthetic hazy dataset. (a) Hazy image, (b) Ground Truth, (c) FFA [2], (d) GDN [3], (e) IA-YOLO [12], (f) Dehamer [5], (g) MSBDN [4], (h) Ours.

First, based on the synthetic hazy data selected in ITS, this study applied the dehazing methods mentioned earlier and the innovative dehazing method proposed in this paper respectively, and carried out in-depth analysis and detailed comparison of the processed results. The result images of various methods are shown in Fig 3.

It can be observed that works such as FFA-Net [2], GDN [3], Dehamer [5], and MSBDN [4] all exhibit excellent dehazing effects when facing the datasets they have trained on. While compared with the trained models, our work also achieves a satisfactory degree in terms of color restoration, clarity, and detail preservation.

**Fig 4:**
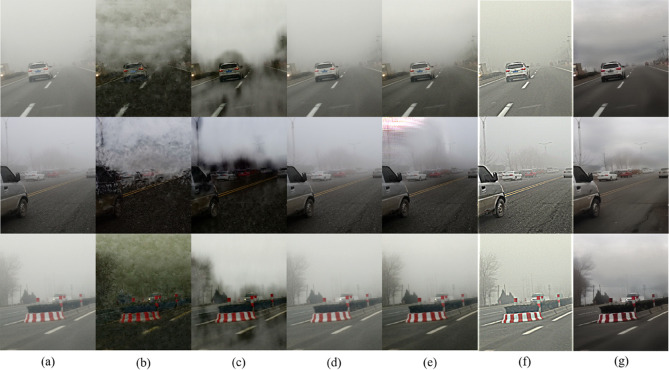
Subjective comparison on the RTTS real-world hazy dataset. (a) Hazy image, (b) FFA [2], (c) GDN [3], (d) IA-YOLO [12], (e) Dehamer [5], (f) MSBDN [4], (g) Ours.

Next, based on the real hazy data (without corresponding real-clear images) selected in RTTS, we conduct an analysis and comparison of the processed results. The dehazing result images of various methods are shown in Fig 4.

As shown in Fig 4, FFA [2] and GDN [3] exhibit severe noise artifacts and ghosting on the RTTS real-world hazy dataset, which significantly affect the dehazing results. IA-YOLO [12], on the other hand, displays noticeable graininess and overexposure, with clear white borders on both sides. Although Dehamer [5] and MSBDN [4] remove haze to some extent, they only eliminate haze that is relatively close, with little effect on the haze in the distance. Additionally, during the experiment, we observed that MSBDN [4] sometimes introduced blurry spots and scale distortion. In the real hazy scenes and under the condition of lacking reference images, the above-mentioned models expose relatively obvious limitations. Their dehazing effects are not satisfactory, and it is difficult for them to effectively deal with complex and changeable scenes, reflecting insufficient adaptability to different scenes. In contrast, the method proposed in this study demonstrates significant advantages.

**Fig 5:**
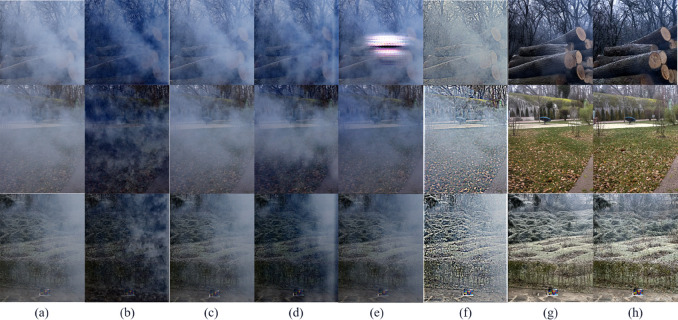
Subjective comparison of light hazy images in the NH-HAZE artificially simulated hazy dataset. (a) Hazy image,(b) FFA [2], (c) Dehamer [5], (d) GDN [3], (e) MSBDN [4], (f) IA-YOLO [12], (g) Ours, (h) Ground truth.

**Fig 6:**
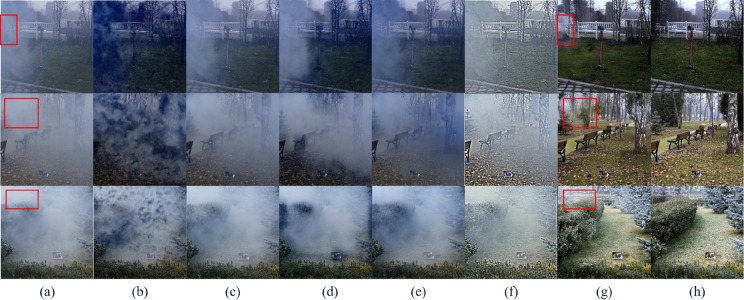
Subjective comparison of dense hazy images in the NH-HAZE artificially simulated haze-filled dataset. (a) Hazy image,(b) FFA [2], (c) Dehamer [5], (d) GDN [3], (e) MSBDN [4], (f) IA-YOLO [12], (g) Ours, (h) Ground truth.

Finally, we conduct a comparative experiment on the NH-HAZE dataset. This dataset uses a professional haze generator to simulate real-world hazy scene conditions, generating images with non-uniform haze and forming image pairs with the corresponding haze-free images. We analyze and compare the processed results. The dehazing result images of various methods are shown in Figs 5 and 6.

As can be observed from Fig 5, for the dehazing of non-uniform hazy images, our method achieves a higher degree of scene restoration. It can recover high-quality images with natural visual features and clear content structures from low-quality, blurry hazy images. However, the models that have not been trained on this dataset perform poorly in dehazing when facing these hazy images.

We can observe from Fig 6 that for the dehazing of dense hazy images, apart from the completely invisible parts of the image caused by the dense haze (marked by the red box), our method can still restore clear, sharp, high-contrast and detailed haze-free images.

**Fig 7:**
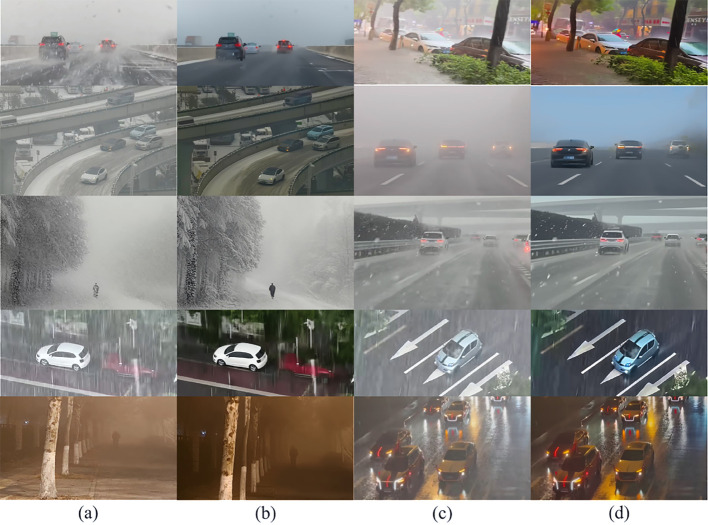
Demonstrates HazeDiff’s adaptability in extreme scenarios. Including heavy rain, blizzards, thick fog, nighttime haze, and other challenging conditions, Images (a) and (c) are captured under various adverse weather conditions; images (b) and (d) show the results after image restoration using HazeDiff.

We conduct evaluations on more diverse real-world datasets and challenging scenarios to demonstrate the robustness of the method. As shown in the Fig 7.

Fig 7 further demonstrates the adaptability of HazeDiff in extreme scenarios, including heavy rain, heavy snow, thick fog, and night haze, highlighting the robustness of HazeDiff in other extremely bad weather conditions.

**Fig 8:**
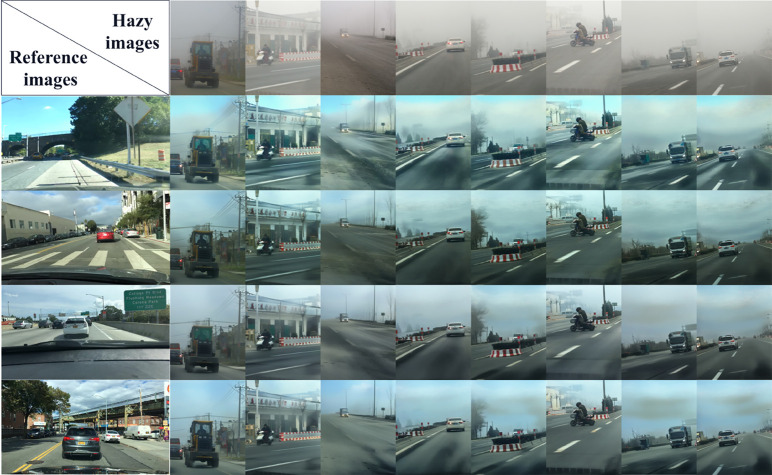
Supplementary experiments were conducted using reference images with varying scene configurations and illumination conditions.

Meanwhile, we conducted supplementary experiments using reference images under different scenes and lighting conditions. The visualization result is shown in Fig 8.

It can be seen from Fig 8 that when the similarity between the reference image and the image to be defogged is very low, although the defogging result leans towards the reference image in terms of color distribution, the key structures (such as building contours and road signs) are still completely retained, prioritizing the integrity of the structure and avoiding the risk of distortion. It has been verified that our method can still achieve a good defogging effect when the similarity between the reference image and the image to be defogged is relatively low.

**Fig 9:**
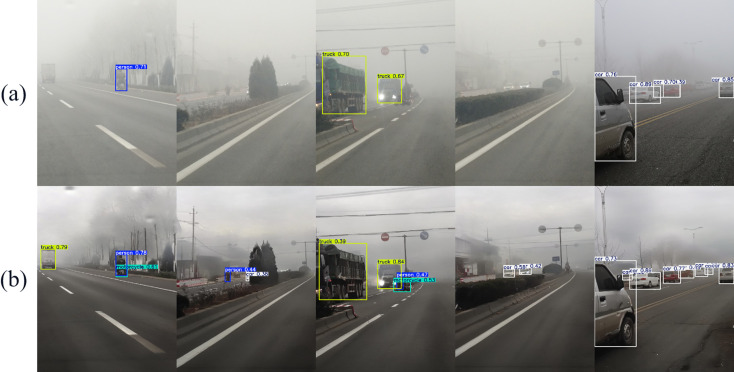
Comparison of visualization results of object detection on the real-world hazy dataset RTTS. (a) Hazy image, (b) dehazed image.

To more intuitively demonstrate the effectiveness of our dehazing method, we compare the visualization results of YOLOv8 detecting the original image and the dehazed image, as shown in Fig 9.

We can observe that the images dehazed by our method produce higher confidence detection results with fewer missed instances. These examples demonstrate that under real-world hazy conditions, our method can adaptively output clearer images with sharper edges, thereby helping the object detection model accurately recognize and locate objects in hazy images.

#### Quantitative analysis of various data in the experiment.

In this section, we carried out a quantitative comparison and ranked various comparison methods, including ours. As previously stated, in the research of image dehazing without Ground truth reference, we employed the reference-free metrics NIQE [32] and BRISQUE [33] to assess the dehazing results of the real hazy images selected from RTTS, so as to make up for the limitations of PSNR/SSIM. All results are reported as the mean, standard deviation (SD) from five independent experiments to ensure the stability and statistical reliability of performance evaluation. The SD reflects the variability in model performance across different image samples, while the 95% confidence interval (CI) indicates the plausible range of values for each metric. For the evaluation metrics adopted in this table NIQE and BRISQUE lower values correspond to superior image quality, demonstrating enhanced dehazing performance. The detailed results are shown in Table 2.

**Table 2:** Performance comparison on RTTS test set.

Method	NIQE↓	SD	95%CI	BRISQUE↓	SD	95%CI
FFA [2]	20.362	0.95	[19.99,20.73]	38.420	1.10	[37.94,38.90]
GDN [3]	11.797	0.68	[11.53,12.06]	19.915	0.75	[19.63,20.20]
IA-YOLO [12]	12.078	0.74	[11.81,12.35]	18.836	0.72	[18.59,19.08]
Dehamer [5]	10.350	0.60	[10.14, 10.56]	14.985	0.68	[14.72,15.25]
MSBDN [4]	9.560	0.48	[9.37,9.75]	16.346	0.62	[16.09,16.60]
Hazy Image	10.705	0.55	[10.50,10.91]	17.013	0.70	[16.74,17.29]
Ours	**8.561**	0.44	[8.38,8.74]	**11.346**	0.51	[11.14,11.56]

*Note.* Since there are no reference images in the RTTS dataset, we use the reference-free image evaluation metrics NIQE and BRISQUE. Best results in **bold**.

The quantitative evaluation results of our method and other recent methods are shown in the above table. As we can see, our method achieves the lowest NIQE and BRISQUE values, outperforming other dehazing methods. It is evident that the dehazing effect of the method proposed in this paper is the most satisfactory for hazy images.

We conduct an evaluation and comparison of the de-hazed images from the NH-HAZE dataset and the ITS subset within the RESIDE [29] dataset. All results are reported as the mean, SD from five independent experiments to ensure the stability and statistical reliability of performance evaluation. The SD reflects the variability in model performance across different image samples, while the 95% CI indicates the plausible range of values for each metric. For the evaluation metrics adopted in this table PSNR and SSIM higher values correspond to superior image quality, demonstrating enhanced dehazing performance.

By analyzing Table 3, our proposed model achieved the best values for all SSIM and PSNR metrics. The experimental results indicate that, compared with other dehazing methods, the dehazing effect of the proposed method is better, and it has good structural awareness and color preservation capabilities.

**Table 3:** Transposed comparison of dehazing methods on NH-HAZE and RESIDE datasets.

Method	NH-HAZE			RESIDE		
	PSNR↑	SD	95% CI	PSNR↑	SD	95% CI
FFA	17.8229	0.65	[17.56, 18.09]	28.1717	0.52	[27.95, 28.39]
GDN	20.6926	0.74	[20.40, 20.98]	29.1742	0.48	[28.98, 29.37]
IA-YOLO	26.8653	0.88	[26.51, 27.22]	28.0068	0.60	[27.75, 28.26]
Dehamer	23.6725	0.70	[23.41, 23.93]	27.8264	0.58	[27.60, 28.05]
MSBDN	22.6947	0.64	[22.46, 22.92]	29.3437	0.42	[29.18, 29.50]
Ours	**29.6970**	0.62	[29.45, 29.94]	**30.3932**	0.39	[30.24, 30.54]
**Method**	**NH-HAZE**			**RESIDE**		
	SSIM↑	SD	95% CI	SSIM↑	SD	95% CI
FFA	0.2264	0.014	[0.221, 0.232]	0.7771	0.019	[0.770, 0.784]
GDN	0.3809	0.018	[0.374, 0.388]	0.8226	0.015	[0.817, 0.828]
IA-YOLO	0.4458	0.020	[0.437, 0.454]	0.5922	0.022	[0.584, 0.600]
Dehamer	0.2618	0.016	[0.256, 0.268]	0.4938	0.020	[0.486, 0.501]
MSBDN	0.3064	0.018	[0.299, 0.313]	0.8908	0.014	[0.886, 0.896]
Ours	**0.7526**	0.012	[0.299, 0.313]	**0.9122**	0.010	[0.908, 0.916]

*Note.* Since haze-free reference images are available in both the NH-HAZE and RESIDE(ITS) datasets, we employ SSIM and PSNR as evaluation metrics. Best results in **bold**.

#### Comparison with the state-of-the-art object detection methods under hazy conditions.

We recognize that in real-world applications, image dehazing is often used as a preprocessing step for higher-level vision tasks such as object detection. Therefore, we use the object detection performance on dehazed images as a no-reference, task-specific evaluation standard to assess the dehazing results of real-world hazy images that lack clean, real references. Specifically, we follow the method in [20] and adopt a task-driven evaluation approach, using several state-of-the-art hazy image object detection models to detect images in the RTTS dataset and obtain the mAP, including DS-Net [35], IA-YOLO [12], YOLOX [36], SWDA [37], TogetherNet [38], MS-DAYOLO [39], and our dehazed images detected by YOLOv8. We rank all algorithms based on the mAP(%) results to evaluate the impact of the dehazing algorithm on the performance of downstream tasks. The mAP results are shown in Table 4.

**Table 4:** Performance comparison on RTTS test set.

Method	Bicycle	Bus	Car	Motor-cycle	Person	mAP(%)
DS-Net [35]	18.0	15.4	46.1	15.2	68.8	32.7
IA-YOLO [12]	35.3	13.6	41.1	21.0	67.3	35.7
SWDA [37]	35.6	18.1	48.2	26.3	53.4	36.3
YOLOX [36]	54.4	17.8	63.9	36.9	65.7	47.7
MS-DAYOLO [39]	44.1	36.5	69.7	37.5	74.2	52.4
TogetherNet [38]	57.3	37.0	75.3	55.4	82.7	61.6
Ours	55.8	63.6	80.7	69.8	65.4	**67.1**

*Note.* Object detection accuracy across categories. Best results in **bold**. All values in percentages.

As shown in Table 4, the dehazed RTTS dataset using our method achieves better results in object detection compared to the currently advanced hazy weather object detection algorithms. Specifically, images from the RTTS dataset, after being dehazed with the proposed method and then processed through YOLOv8 for object detection, achieved the highest mAP. This indicates that our method effectively removes haze while improving image quality, making objects in the image clearer and more distinguishable, thereby enhancing the accuracy and robustness of object detection. This further demonstrates the reliability and effectiveness of our method in real-world scenarios, providing an effective solution for image dehazing.

We conducted rigorous testing on the NVIDIA RTX 4090 (24GB VRAM) GPU, with key metrics shown in Table 5. Compared to SD, our method achieves faster single-image inference time and lower VRAM usage, enabling efficient inference with significant computational cost advantages. In future work, we plan to employ parameter-efficient fine-tuning techniques such as LoRA to reduce the parameter count of UNet, further optimizing the computational efficiency of our method.

**Table 5:** Performance comparison on RTTS test set.

Indicator	HazeDiff	SD Baseline	Relative Advantage
Single Image Inference Time (512×512)	2.1 seconds	2.3 seconds	+9.5%
Peak Video Memory Usage	17.8GB	18.2GB	+2.2%
Sampling Steps	50	50	–

### Ablation experiments

In this section, we conducted several ablation studies to analyze the effectiveness of the proposed HazeDiff on real-world hazy weather datasets. These studies include our model HazeDiff, HazeDiff (without SRM), HazeDiff (without PFI)and the determination of the final parameter settings.

**Fig 10:**
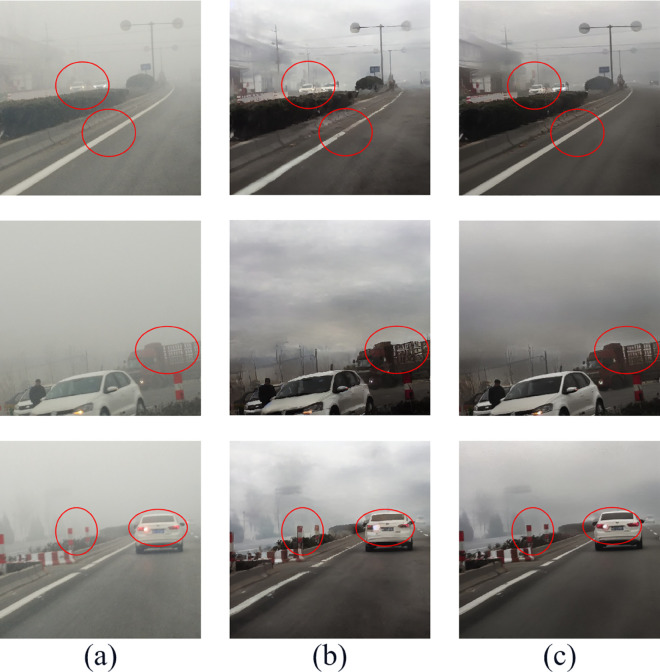
Comparison of content structure preservation in original hazy images between HazeDiff and HazeDiff (without SRM). (a) Hazy images, (b) HazeDiff, (c) HazeDiff (without SRM).

We applied the same reference image to guide the image dehazing for both HazeDiff and HazeDiff (without SRM), and the experimental results are shown in Fig 10.

It can be observed that, through SRM, key structural features in hazy images, such as object contours and relative spatial relationships, are better preserved during the dehazing process. For instance, the shapes of buildings and the appearance of vehicles are more effectively presented in the final dehazed images.

**Fig 11:**
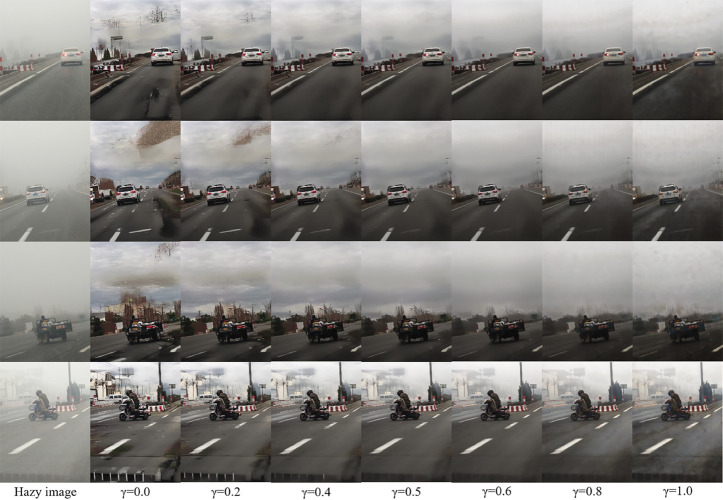
Analysis of the mixing degree parameter, we adjust the dehazing strength by changing the value of *γ.*

We also conducted experiments on the blending degree parameter *γ* in the model, quantitatively evaluating the generated images under different parameters to explore the optimal value for addressing the potential blurring of original structural details in hazy images during the generation process. The quantitative analysis results are shown in Table 6 and the visualization results are shown in the Fig 11.

We observed that when the mixing degree parameter *γ* is less than 0.4, although the dehazing effect is significant and the colors are clear and bright, the structural content of the original image is not well preserved. This results in generated images that differ greatly in content from the original hazy images, failing to accurately convey their semantic information and visual effects. When the mixing degree parameter *γ* is greater than 0.6, while the structure of the hazy images is well preserved, the haze is not effectively removed. However, when *γ* is between 0.4 and 0.6, the haze can be reasonably removed without introducing additional noise, while retaining the structural content of the hazy images. Therefore, we selected a mixing degree parameter of γ=0.5, which lies between 0.4 and 0.6.

**Fig 12:**
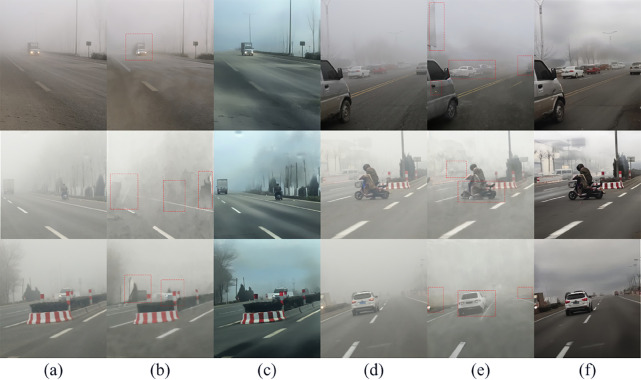
Comparison of images generated by Hazediff and Hazediff with the PFI removed.

To validate the effectiveness of the proposed PFI method, we performed haze removal using Hazediff with the PFI removed. The visualization results are shown in the Fig 12. Columns (a) and (d) present the hazy input images requiring processing. Panels (b) and (e) demonstrate the dehazing results from the PFI-ablated Hazediff variant, where structural distortions (highlighted in red dashed boxes) manifest in critical regions. In contrast, panels (c) and (f) exhibit the restoration outputs from the full Hazediff framework with intact PFI.

The results revealed that in the absence of PFI to effectively guide the content images, the generated images exhibited significant randomness. This forced the model to autonomously interpret the definition of “haze removal,” leading to severe image degradation with substantial loss of fine details, while failing to achieve the intended dehazing effect. These findings demonstrate that the proposed PFI provides superior guidance for pixel-level transformation of content images.

To comprehensively assess the individual contributions and parameter sensitivity of the PFI and SRM modules, we conducted quantitative experimental analyses, as shown in Table 6.

**Table 6:** Ablation study on the mixing parameter *γ* using RESIDE-ITS and RTTS datasets. Values are reported as mean ± SD. For PSNR and SSIM, higher is better; for NIQE and BRISQUE, lower is better.

γ	RESIDE-ITS		RTTS	
	PSNR ↑	SSIM ↑	NIQE ↓	BRISQUE ↓
0.0	26.75 ± 0.45	0.846 ± 0.012	9.43 ± 0.47	13.62 ± 0.66
0.2	28.45 ± 0.42	0.880 ± 0.011	8.86 ± 0.45	12.36 ± 0.58
0.4	29.85 ± 0.40	0.902 ± 0.010	8.64 ± 0.43	11.73 ± 0.53
**0.5**	**30.90 ± 0.38**	**0.918 ± 0.009**	**8.52 ± 0.42**	**11.27 ± 0.51**
0.6	29.10 ± 0.40	0.893 ± 0.011	8.82 ± 0.44	11.93 ± 0.55
0.8	27.35 ± 0.44	0.860 ± 0.012	9.25 ± 0.46	12.87 ± 0.60
1.0	25.92 ± 0.49	0.827 ± 0.014	9.78 ± 0.48	13.95 ± 0.65

**Table 7:** Performance comparison of different model versions on image quality metrics.

Model Version	Image Quality Metrics			
	PSNR ↑	SSIM ↑	NIQE ↓	BRISQUE ↓
	Mean ± SD (95% CI)	Mean ± SD (95% CI)	Mean ± SD (95% CI)	Mean ± SD (95% CI)
Baseline	17.85 ± 0.64	0.2302 ± 0.015	20.31 ± 0.70	38.07 ± 0.95
	[17.61, 18.09]	[0.224, 0.236]	[20.05, 20.57]	[37.71, 38.43]
+PFI	28.05 ± 0.48	0.8725 ± 0.010	9.01 ± 0.43	14.64 ± 0.66
	[27.86, 28.24]	[0.868, 0.877]	[8.85, 9.17]	[14.39, 14.89]
+PFI + SRM	30.39 ± 0.39	0.9122 ± 0.010	8.56 ± 0.44	11.35 ± 0.51
	[30.22, 30.56]	[0.908, 0.916]	[8.38, 8.74]	[11.14, 11.56]

The experimental results demonstrate that the PFI module plays a decisive role in improving image reconstruction quality. By incorporating the SRM module, the experimental data are further optimized and achieve optimal values, thereby comprehensively assessing the individual contributions of both the PFI and SRM.

## Conclusion

In this paper, we propose a Diffusion Model-based method, HazeDiff, to address several challenging image dehazing problems. Specifically, we propose PFI, which extracts pixel-level features from the reference image and injects them into the latent noise of the dehazed image, guiding the diffusion model to generate the dehazed image in an unsupervised manner. Next, we design an attention-based SRM to focus on the key regions and details of hazy images, addressing the issue where diffusion models guided by reference images may blur the original structural details of the hazy image during the generation process. Compared to previous dehazing methods, HazeDiff has stronger generalization ability and does not require a large number of paired hazy-clear images from the same scene for training. Extensive experiments show that our HazeDiff outperforms other state-of-the-art dehazing methods, achieving the best results.
